# The Role of Dopaminergic VTA Neurons in General Anesthesia

**DOI:** 10.1371/journal.pone.0138187

**Published:** 2015-09-23

**Authors:** Xuelong Zhou, Yin Wang, Chenjing Zhang, Min Wang, Mei Zhang, LiNa Yu, Min Yan

**Affiliations:** 1 Department of Anesthesiology, The Second Affiliated Hospital, School of Medicine, Zhejiang University, Hangzhou, 310000, China; 2 Department of Anesthesiology, The Tai Zhou People’s Hospital, Taizhou, 225300, China; 3 Department of Gastroenterology, The Second Affiliated Hospital, School of Medicine, Zhejiang University, Hangzhou, 310000, China; 4 Jiangsu Key Laboratory of Anesthesiology, Xuzhou Medical College, Xuzhou, 221000, China; Massachusetts General Hospital, UNITED STATES

## Abstract

Recent studies have demonstrated that the central dopaminergic system is implicated in the mechanism underlying general anesthesia. Here, we investigated whether dopaminergic ventral tegmental area (VTA) neurons participate in general anesthesia. Dopaminergic VTA neurons were selectively ablated from male Sprague Dawley rats via the bilateral infusion of 6-hydroxydopamine (6-OHDA) into the VTA. Two weeks after infusion, the number of dopaminergic neurons in the bilateral VTA was markedly reduced in the 6-OHDA-treated rats compared with the vehicle-treated rats. These bilateral VTA lesions significantly prolonged the recovery time for propofol but did not significantly alter its onset time or 50% effective dose (ED50) value. In addition, the anesthetic responses to isoflurane and ketamine were unaffected by the VTA lesions. Our findings suggested that dopaminergic VTA neurons might be involved in the emergence from propofol anesthesia.

## Introduction

Accumulating evidence indicates that the endogenous sleep and/or arousal pathways in the brain are involved in the induction and maintenance of general anesthesia [1,2]. General anesthetics exert their hypnotic effect by potentiating the sleep pathways or suppressing the arousal pathways. The modulation of the activity of the sleep and arousal pathways may facilitate or prolong general anesthetic-induced unconsciousness.

The dopaminergic system, a component of the central nervous system (CNS), is involved in a variety of physiological and neurological processes, such as movement, reward, sleep and arousal [3]. Two recent studies have shown that central dopaminergic pathways are involved in the modulation of general anesthesia. The activation of the central dopaminergic D1 receptor decreases the emergence time and induces the recovery from isoflurane anesthesia [4]. Electrical stimulation of the ventral tegmental area (VTA), a major dopamine nuclei in the brain, also induces reanimation from general anesthesia [5]. These findings suggest a potential functional role of the central dopaminergic pathways in general anesthesia. However, because of the limitations of the approaches used in previous studies (the small size of the VTA and the presence of other types of neurons make precision stimulations challenging), the conclusion that dopaminergic VTA neurons participate in general anesthesia should be drawn carefully. Furthermore, whether dopaminergic VTA neurons are involved in modulating the hypnotic effects of general anesthetics remains unclear. In the present study, using 6-hydroxydopamine (6-OHDA) [6], we selectively lesioned the dopaminergic neurons in the VTA of rats and assessed the effects of this treatment on the properties of general anesthetics.

## Materials and Methods

### 2.1. Animals

The experiments were performed on 46 adult male Sprague Dawley rats (8-12-week-old, weighing 220–250 g), which were obtained and housed in the Experimental Animal Center of Zhejiang University. All experimental procedures were approved by the animal care committee of Zhejiang University.

### 2.2. VTA lesioning

The lesioning of dopaminergic VTA neurons was conducted as described previously [6]. After anesthesia (sodium pentobarbital, 50 mg/kg, intraperitoneally (i.p.)), the rats underwent bilateral infusion of 6-hydroxydopamine (6-OHDA; 3 μg/0.5 μl) or vehicle into the VTA (from bregma: anteroposterior, 5.5 mm; mediolateral, ±0.5 mm; dorsoventral, -7.8 mm). Desipramine (20 mg/kg) was administered i.p. 1 h later to protect the noradrenergic neurons [6]. After a 2-week recovery period, the rats were exposed to anesthetics to determine their anesthetic responses. In order to minimize the number of animals used, each rat was tested for two or three general anesthetics in a random order and separated by 1 week.

### 2.3. Anesthetic response

We determined the anesthetic responses to isoflurane, propofol, and ketamine, as demonstrated by the 50% effective concentration (EC50) or dose (ED50) and the times of the onset of and recovery from each anesthetic. These procedures have been described previously [7].

Isoflurane was delivered into a plexiglas observation chamber by a calibrated vaporizer with 1 l/min flow of pure oxygen, and leaked through small holes on the other side. The EC50 or ED50 of the anesthetics was determined as follows. The rats were exposed to isoflurane, propofol, and ketamine at an incremental concentration or dose (isoflurane: 0.625%, increased by 0.125% at minimum 15-min intervals; propofol or ketamine (i.p.): 40 mg/kg, increased by 20 mg/kg at 10-min intervals), and the concentration or dose resulting in the loss of the righting reflex (LORR) on three successive trials was recorded. The dose-LORR data were then curve-fitted using the following equation: Y = Y_min_ + (Y_max_—Y_min_) / [1 + 10^log(ED50—X)^*^m^], where Y is the percentage of the population anesthetized, X is the logarithmic drug dose, m is the slope parameter; and ED50 is the drug dose resulting in the half-maximal effect [8].

The times of onset of and recovery from the anesthetic were determined as follows. The rats were exposed to isoflurane, propofol, and ketamine at a single concentration or dose corresponding to the ED95 based on the dose-LORR curve (isoflurane: 1.375%; propofol: 140 mg/kg (i.p.); and ketamine (i.p.): 180 mg/kg), and then, the time of onset was recorded. For isoflurane, the rats were exposed to room air at 30 min after exposure, the time to recovery was then determined. Throughout the experiment, the body temperature was maintained at 37±0.5°C by a warming pad.

### 2.4. Immunohistochemistry

After all experiments, the brains were dissected from paraformaldehyde-perfused rats, dehydrated in sucrose, and sectioned into slices. The immunohistochemistry procedure was performed using a detection kit as described previously [9]. The dopaminergic or GABAergic VTA neurons were identified using the anti-Tyrosine Hydroxylase (TH) or anti-glutamic acid decarboxylase (GAD 65/67) primary antibody, respectively. The ten sections containing the greatest number of positively immunostained neurons were selected, and the number of immunopositive neurons in the central region of the VTA or the substantia nigra (SN) was recorded.

### 2.5. Statistical Analysis

The data are presented as the means±SD or as the medians±inter-quartile range. The number of TH- or GAD 65/67-immunoreactive cells in the VTA and the EC50 or ED50 values were analyzed using the unpaired Student’s t test. The times of onset of and recovery from the anesthetic were analyzed using a non-parametric (Mann—Whitney) test. All analyses were performed using SPSS version 13.0. A value of *p*<0.05 was considered to be significant.

## Results

### 3.1. Dopaminergic VTA neuron lesioning

**Fig 1:**
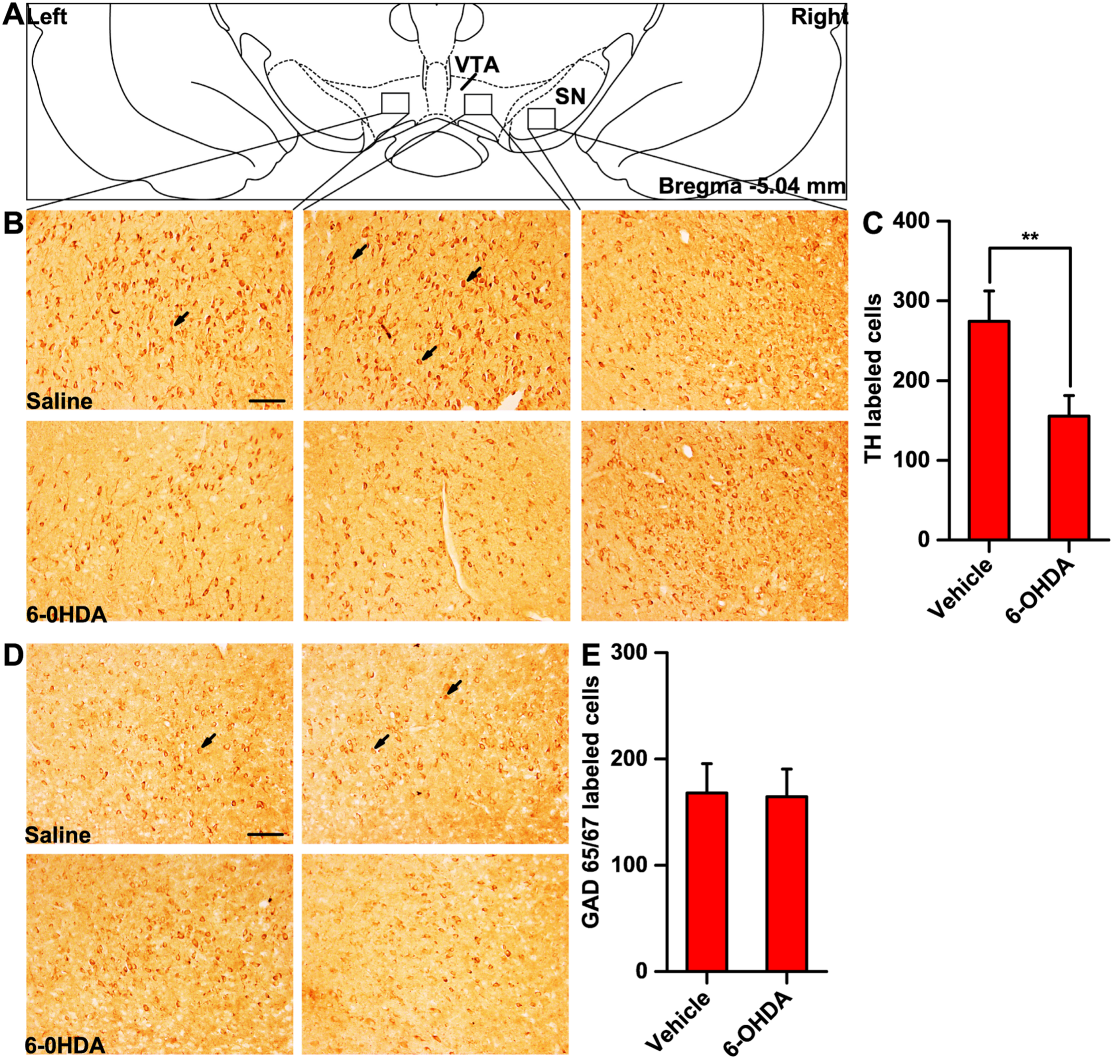
The effects of the bilateral infusion of 6-OHDA into the VTA on the number of dopaminergic neurons in the VTA and the SN (B and C) and GABAergic neurons in the VTA (D and E). 6-OHDA: 6-hydroxydopamine; VTA: ventral tegmental area; SN: substantia nigra; TH: tyrosine hydroxylase; GAD: glutamic acid decarboxylase. **p<0.01 *vs*. vehicle; n = 22 for the vehicle group and n = 24 for the 6-OHDA group. Scale bar = 100 μm.

As shown in Fig 1, compared to vehicle infusion, bilateral infusion of 6-OHDA into the VTA significantly reduced (~43%) the number of TH-immunoreactive cells in the VTA (274±38 *vs*. 155±25, *p*<0.01), but no such reduction was observed in the SN. No significant difference in the number of GAD 65/67-immunoreactive cells in the VTA was detected between vehicle and 6-OHDA infusion (168±27 *vs*. 164±26, *p*>0.05).

### 3.2. Anesthetic response to isoflurane

**Fig 2:**
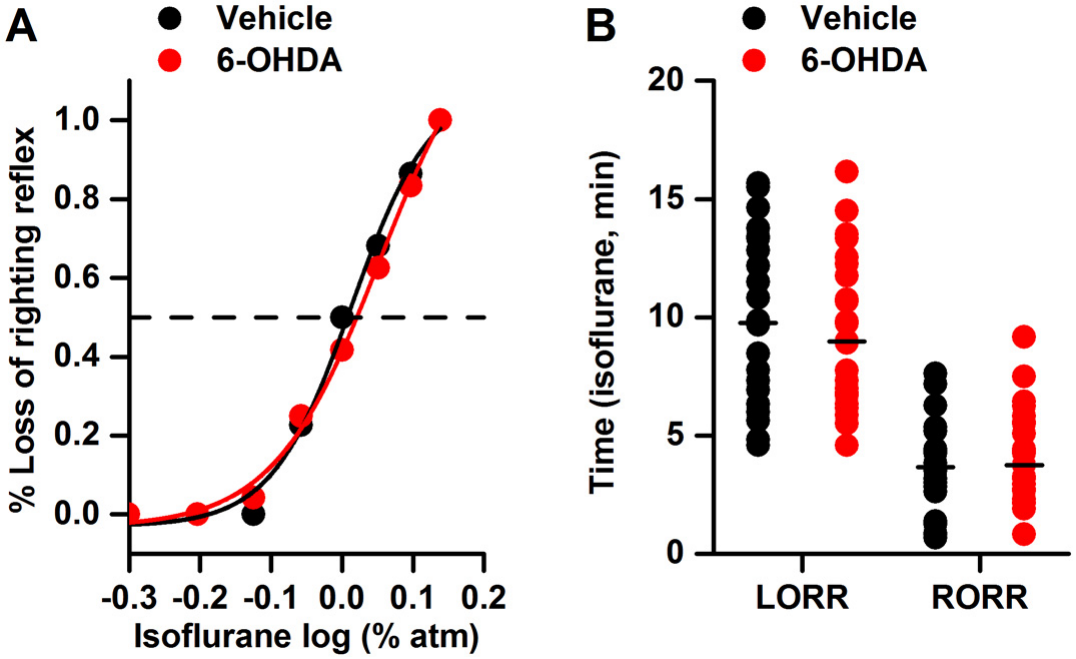
The effects of the bilateral lesioning of the dopaminergic VTA neurons on the isoflurane ED50 value and the times of onset of and recovery from isoflurane anesthesia. 6-OHDA: 6-hydroxydopamine; LORR: loss of the righting reflex; RORR: return of the righting reflex. *P*>0.05 *vs*. vehicle; n = 22 for the vehicle group and n = 23 for the 6-OHDA group.

As shown in Fig 2, compared to the control treatment, bilateral VTA lesioning exerted no significant effect on the ED50 value of isoflurane (1.03±0.14% *vs*. 1.13±0.26%, *p*>0.05). In addition, compared to the control conditions, neither the time of onset of nor recovery from isoflurane anesthesia was affected by the VTA lesions [onset: 9.77 (6.77–13.35) min *vs*. 9.00 (6.70–12.25) min, *p*>0.05; recovery: 3.67 (1.37–4.61) min *vs*. 3.76 (2.67–5.58) min, *p*>0.05].

### 3.3. Anesthetic response to propofol

**Fig 3:**
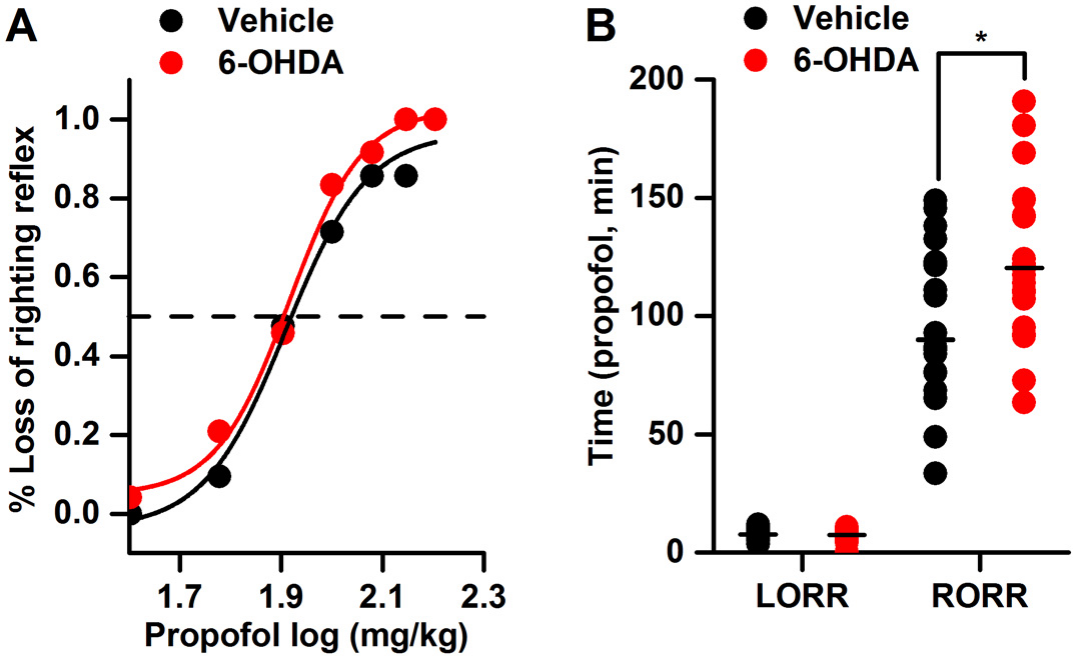
The effects of the bilateral lesioning of the dopaminergic VTA neurons on the propofol ED50 value and the times of onset of and recovery from propofol anesthesia. 6-OHDA: 6-hydroxydopamine; LORR: loss of the righting reflex; RORR: return of the righting reflex. *p<0.05 *vs*. vehicle; n = 21 for the vehicle group and n = 24 for the 6-OHDA group.

As shown in Fig 3, compared to the control treatment, bilateral VTA lesioning significantly prolonged the recovery time of propofol [92.65 (72.27–122.32) min *vs*. 120.2 (107.84–149.25) min, *p*<0.05] but did not affect its ED50 value (81.06±17.57 mg/kg *vs*. 82.33±12.45 mg/kg, *p*>0.05) or the onset time of propofol [7.67 (6.26–10.80) min *vs*. 7.28 (6.00–8.63) min, *p*>0.05].

### 3.4 Anesthetic response to ketamine

**Fig 4:**
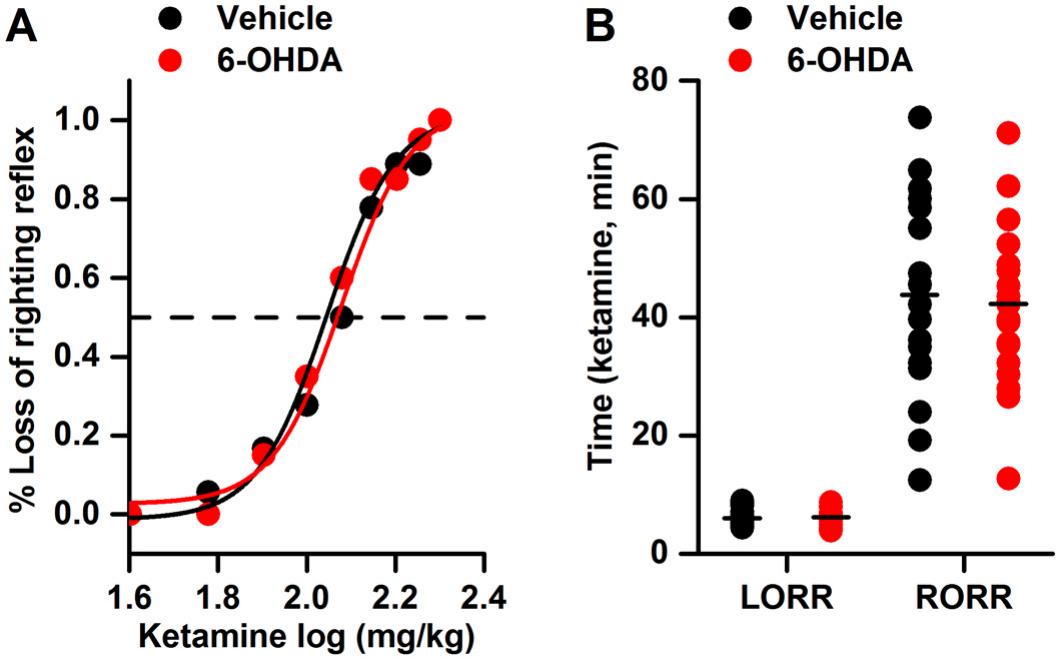
The effects of the bilateral lesioning of the dopaminergic VTA neurons on the ketamine ED50 value and the times of onset of and recovery from ketamine anesthesia. 6-OHDA: 6-hydroxydopamine; LORR: loss of the righting reflex; RORR: return of the righting reflex. *P*>0.05 *vs*. vehicle; n = 18 for the vehicle group and n = 20 for the 6-OHDA group.

As shown in Fig 4, no significant difference in the ketamine ED50 value or the time of onset of or recovery from ketamine anesthesia was detected between the 6-OHDA-lesioned rats and the sham-lesioned rats [ED50: 119.91±17.69 mg/kg vs. 110.84±13.05 mg/kg, *p*>0.05; onset: 5.99 (4.97–8.14) min *vs*. 6.16 (5.90–6.86) min, *p*>0.05; recovery: 43.82 (32.06–58.91) min *vs*. 42.30 (33.06–48.62) min, *p*>0.05].

## Discussion

Although many endogenous sleep or arousal pathways (i.e., the GABAergic [10,11], noradrenergic [12,13], histaminergic [7,14], and cholinergic [15,16] pathways) have been implicated in the mechanism of general anesthesia, few studies have investigated the role of the dopaminergic pathways in general anesthesia. Very recently, using a D1 receptor agonist (chloro-APB), Taylor et al. successfully induced the emergence from isoflurane anesthesia in rats [4]. Subsequently, the identical group reported that electrical stimulation of the VTA, which is a major dopaminergic nucleus in the brain, induces reanimation from general anesthesia [5]. These findings indicated that the central dopaminergic system, particularly the dopaminergic VTA, might be involved in general anesthesia. However, these electrical stimulation approaches are open to interpretation. In rats, the small size of the VTA and the presence of other types of neurons (i.e., GABAergic neurons) in the VTA confound the investigation of dopaminergic VTA neurons. In this study, we examined the effects of treatment of the VTA with 6-OHDA, which selectively ablates dopaminergic neurons [6], on general anesthesia. We found that bilateral VTA lesions only prolonged the recovery time of propofol.

The delayed emergence from propofol anesthesia might be associated with the role of the VTA in mediating arousal [3,17]. By ablating the dopaminergic VTA neurons, the effects of these neurons on arousal were diminished. However, our results revealed that these VTA lesions prolonged the recovery time of propofol, but not isoflurane. These findings are partially different from the results observed in a previous study [5]. In that study, electrical stimulation of the VTA induced reanimation from both isoflurane and propofol anesthesia. We speculate that the differences in the treatment approach between these two studies might account for this discrepancy. Although the authors demonstrated that the electrical VTA stimulation was localized to a small region in the vicinity of the electrode tip and did not stimulate the adjacent areas, electrical stimulation of the VTA might activate other types of neurons (i.e., GABAergic neurons [18]) in the VTA. The activation of non-dopaminergic neurons might affect the emergence from general anesthesia. The central GABAergic neurons have been well demonstrated to modulate general anesthesia [14,19]. In addition, the previous study was performed under a constant anesthetic concentration, so pharmacokinetics of the anesthetics did not play a role in the results. On the other hand, the results of the current study are dependent on pharmacokinetics of the anesthetic drugs. It is possible that the dopaminergic effect on wake up from isoflurane is too small to make a significant difference in the rapid wakeup from isoflurane that is governed by rapid pharmacokinetics. Nonetheless, other factors, such as differences in the experimental procedures, may also underlie the discrepancies between our results and those of the previous study.

In this study, we found that the dopaminergic VTA neurons are critical for the emergence from, but not the induction of, general anesthesia. The time of onset of anesthesia was not affected by VTA lesioning. This finding might have resulted from the dependence of the onset of general anesthesia on the effect of the general anesthetic on brain regions (i.e., the ventrolateral preoptic nucleus) other than the VTA. In fact, several studies have shown that the induction of general anesthesia may involve neuronal circuits different from those responsible for the emergence from anesthesia. The genetically ablation of orexinergic neurons in mice resulted in no change in the anesthetic response of these mice to isoflurane; however, the recovery time was significantly prolonged [20]. Bilateral tuberomammillary nucleus lesions have been reported to delay the emergence from isoflurane anesthesia without affecting the time of onset [7]. These findings suggest that the mechanisms underlying the induction of and the emergence from general anesthesia are not simply mirror opposites. Based on these results, we propose that the dopaminergic VTA neurons might be involved in the modulation of the emergence from propofol anesthesia.

In the present study, we also found that VTA lesioning did not affect the anesthetic response to ketamine. No significant effects on the ED50 value or the times of onset of or recovery from ketamine were detected. The lack of VTA lesion effect on ketamine anesthesia is expected, because of the mechanisms underlying ketamine anesthesia are different from those of isoflurane or propofol [21]. At the molecular level, ketamine blocks N-methyl-Daspartate receptor, while, propofol is an agent that act on GABA_A_ receptor. Contrary to ketamine and propofol, commonly used volatile anaesthetic agents such as isoflurane acts on multiple molecular targets, including GABA_A_ receptors, glycine receptors, glutamate receptors and TREK-1 potassium channels [22].

The findings presented here are very important for understanding the neuronal mechanisms underlying general anesthesia. However, the current study contains several limitations. First, bilateral infusion of 6-OHDA into the VTA only had ~43% reduction of VTA dopamine neurons. Therefore, more than half of the VTA dopamine neuron still intact. This would contributes a lot to the negative results in the present study. A higher dose of 6-OHDA (6 μg) was actually infused in our study. Unfortunately, we found that many rats had an obvious impairment in movement capacity and this impairment significantly affected the time of RORR. Second, although the cumulative intraperitoneal administration of general anesthetics has been widely used in animals to calculate the ED50 [23,24], the complex pharmacokinetics of systemic administration may greatly affect these results. Therefore, the ED50 results should be interpreted with caution. Third, the delay in the recovery from propofol anesthesia might be secondary to the elimination of the drug. Clarifying this issue would require the measurement of the blood propofol concentration.

In summary, under the present experimental conditions, our results showed that dopaminergic VTA neurons are involved in the emergence from propofol anesthesia. This finding may have important implications for exploring the roles and mechanisms of VTA dopamine neurons in general anesthesia.
